# Supplementary material for: Molecular insight into interactions between the Taf14, Yng1 and Sas3 subunits of the NuA3 complex
Source: Nat Commun. 2024 Jun 24;15:5335. doi: 10.1038/s41467-024-49730-y (PMC11196586; doi:10.1038/s41467-024-49730-y)
Supplement: Supplementary file 1 — Supplementary Information [file 41467_2024_49730_MOESM1_ESM.pdf]

Supplementary Information

Molecular insight into interactions between the Taf14, Yng1 and Sas3 subunits of the NuA3 complex

Minh Chau Nguyen, et al.

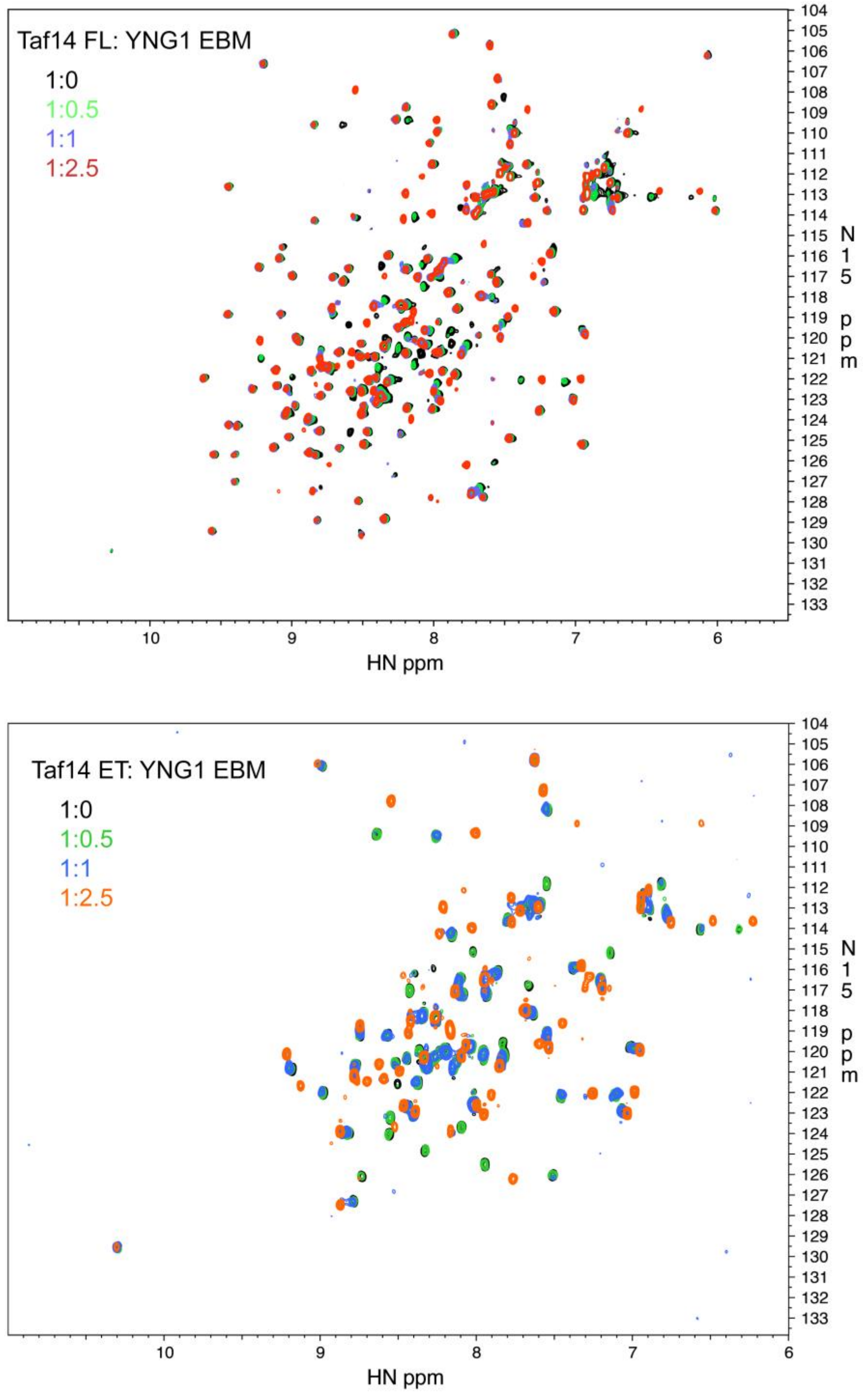

Supplementary Figure 1. Superimposed ^{1}H,^{15}N HSQC spectra of Taf14$_{FL}$ (top) and Taf14$_{ET}$ (bottom) recorded in the presence of increasing amounts of Yng1$_{EBM}$ peptide. The spectra are color coded according to the protein:peptide molar ratio. Related to Figure 1.

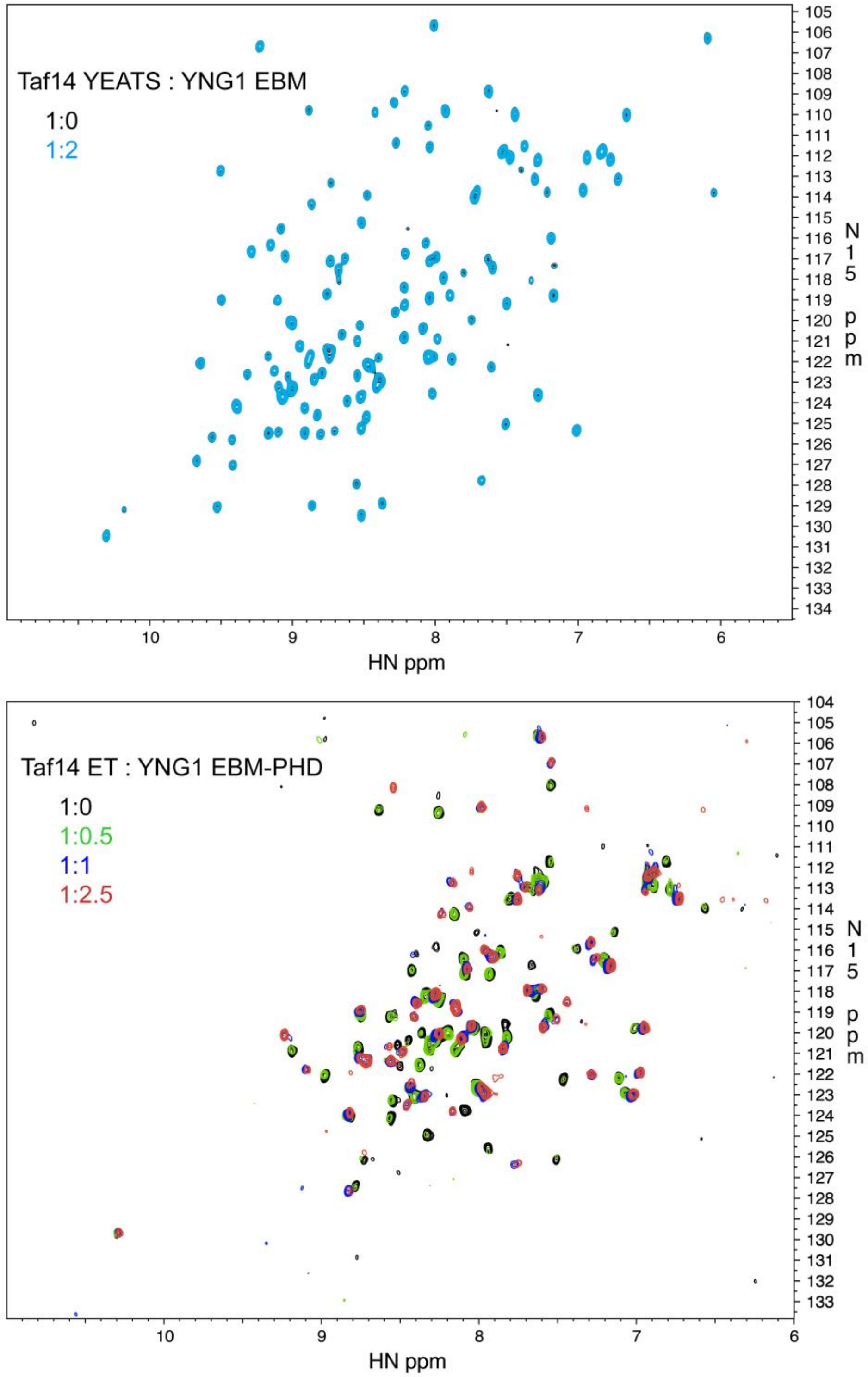

Supplementary Figure 2. Superimposed $^{1}H,^{15}N$ HSQC spectra of Taf14$_{YEATS}$ in the presence of increasing amounts of Yng1$_{EBM}$ peptide (top). Superimposed $^{1}H,^{15}N$ HSQC spectra of Taf14$_{ET}$ in the presence of increasing amounts of purified Yng1$_{EBM-PHD}$ (bottom).The spectra are color coded according to the protein:ligand molar ratio. Related to Figure 1.

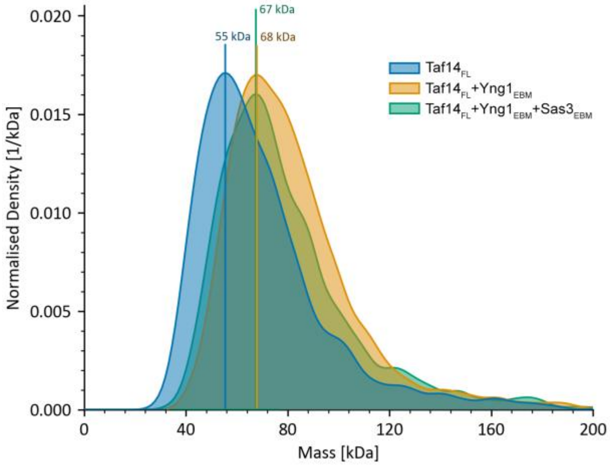

Supplementary Figure 3. Mass photometry analysis of molecular weights for Taf14$_{FL}$ in the apo-state (blue) or in the presence of one molar equivalent of Yng1$_{EBM}$ (orange) or five/one molar equivalents of Sas3$_{EBM}$/Yng1$_{EBM}$ (green) indicating the formation of the dimeric states for apo and EBM-bound Taf14$_{FL}$. Theoretical molecular weights for apo- and Yng1$_{EBM}$-bound Taf14$_{FL}$ are 55 kDa and 58 kDa, respectively. Maxima of the fits estimated by Kernel Density Estimators (KDE) are labeled. Fits were done with a bandwidth of 3. Related to Figures 2 and 6.

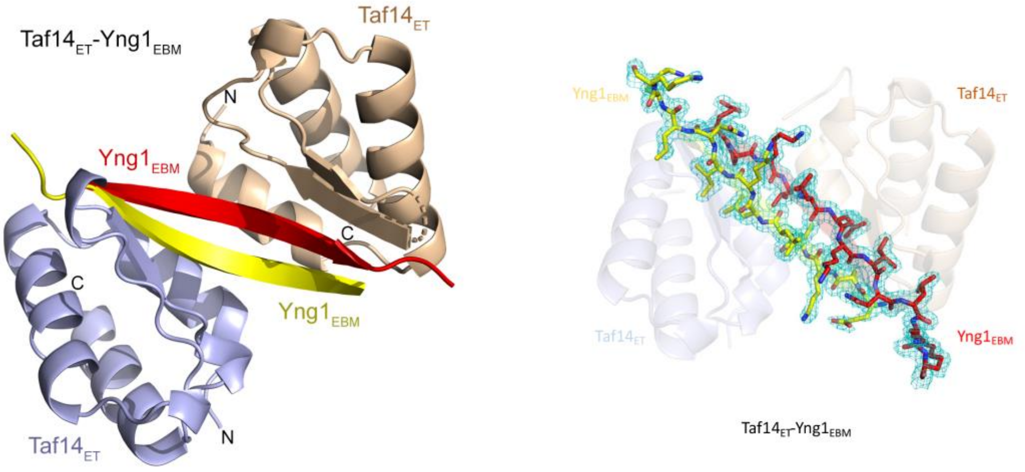

Supplementary Figure 4. (left) Ribbon diagram of the $Taf14_{ET}$-$Yng1_{EBM}$ complex structure. Dimeric $Taf14_{ET}$ is colored light blue (monomer 1) and wheat (monomer 2), and two monomers of dimeric $Yng1_{EBM}$ are colored yellow and red. (right) The 2mFo-DFc map for the $Yng1_{EBM}$ peptide in the complex contoured at the 1σ level. Related to Figure 2.

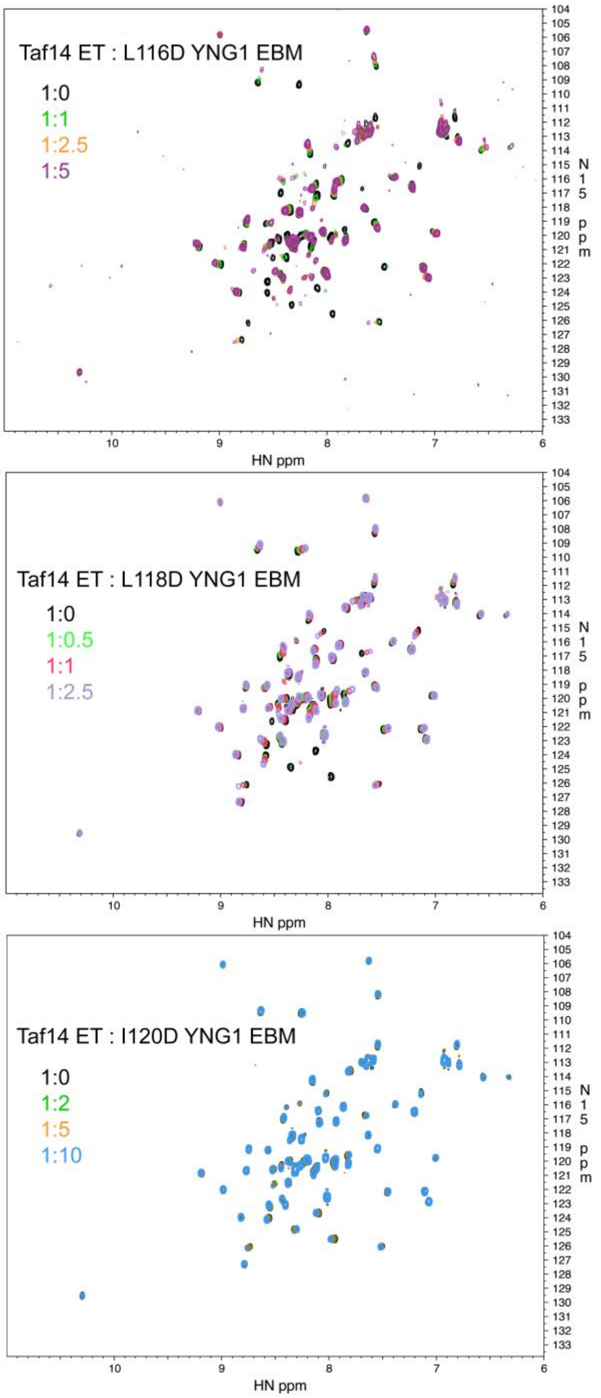

Supplementary Figure 5. Superimposed ^{1}H,^{15}N HSQC spectra of Taf14$_{ET}$ recorded in the presence of increasing amounts of indicated mutated Yng1$_{EBM}$ peptides. The spectra are color coded according to the protein:peptide molar ratio. Related to Figure 3.

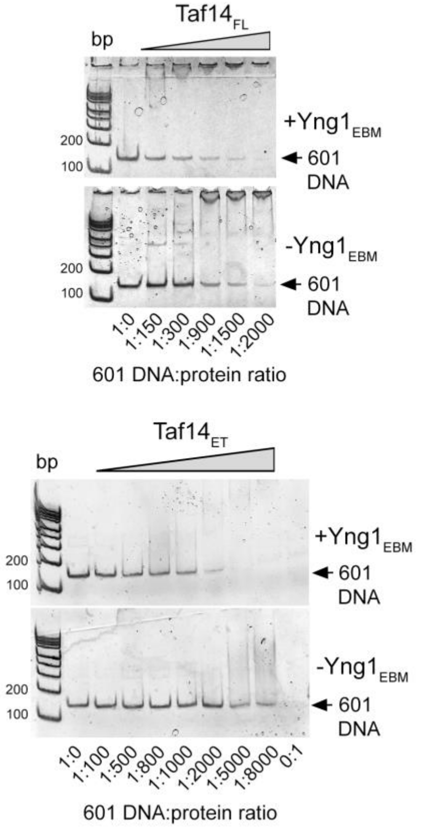

Supplementary Figure 6. EMSAs of 601 DNA in the presence of increasing amounts of $Taf14_{FL}$ (top) or $Taf14_{ET}$ (bottom) +/- $Yng1_{EBM}$. The Taf14 proteins were either preincubated with $Yng1_{EBM}$ at a 1:2.5 molar ratio for a minimum of 1 hour on ice or used without $Yng1_{EBM}$ prior to addition of DNA. Reaction buffer was supplemented with 100 mM NaCl (top) or 25 mM NaCl (bottom). DNA:protein ratio is shown below gel images. Related to Figure 4.

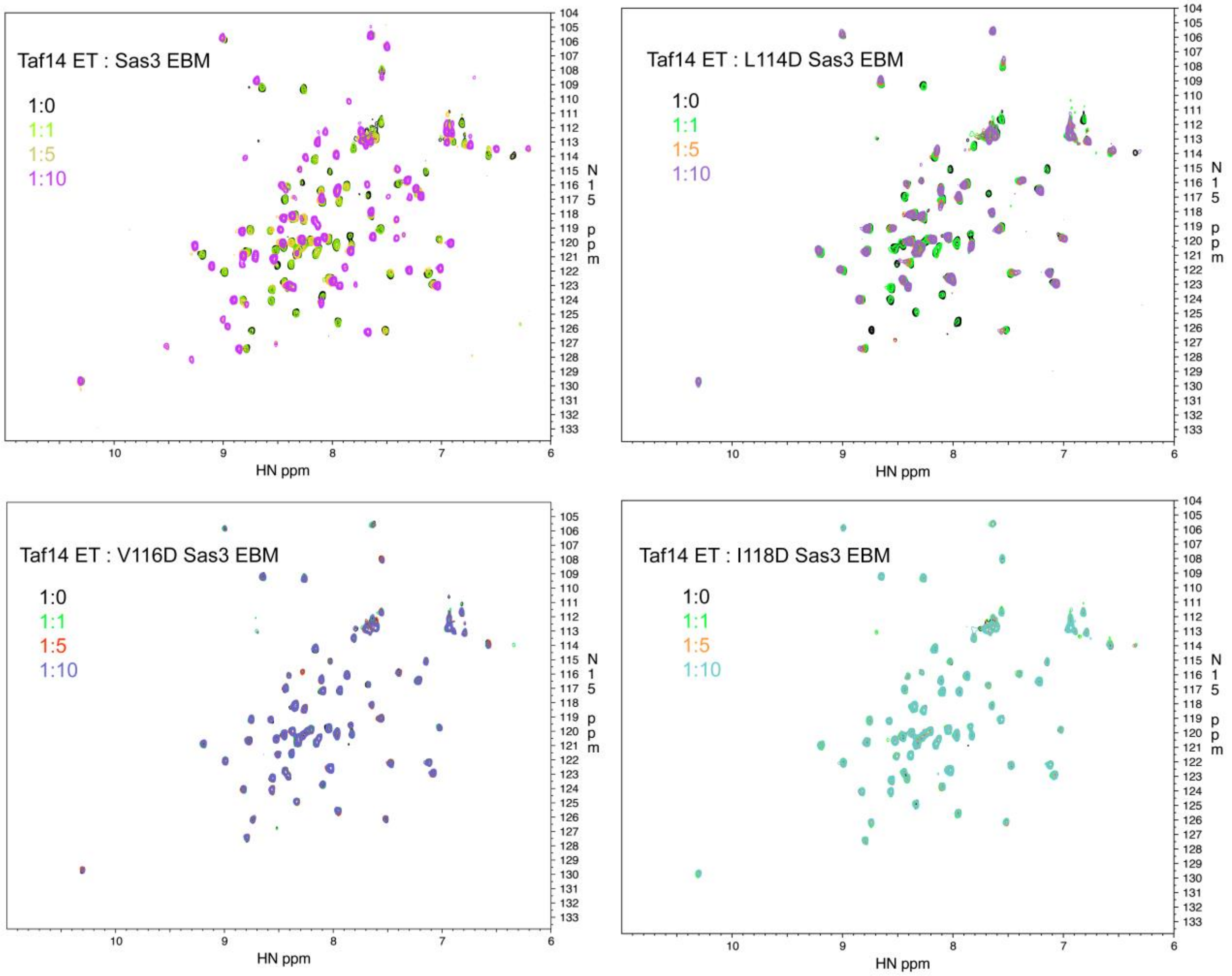

Supplementary Figure 7. Superimposed $^{1}H,^{15}N$ HSQC spectra of $Taf14_{ET}$ recorded in the presence of increasing amounts of indicated $Sas3_{EBM}$ peptides. The spectra are color coded according to the protein:peptide molar ratio. Related to Figure 5.

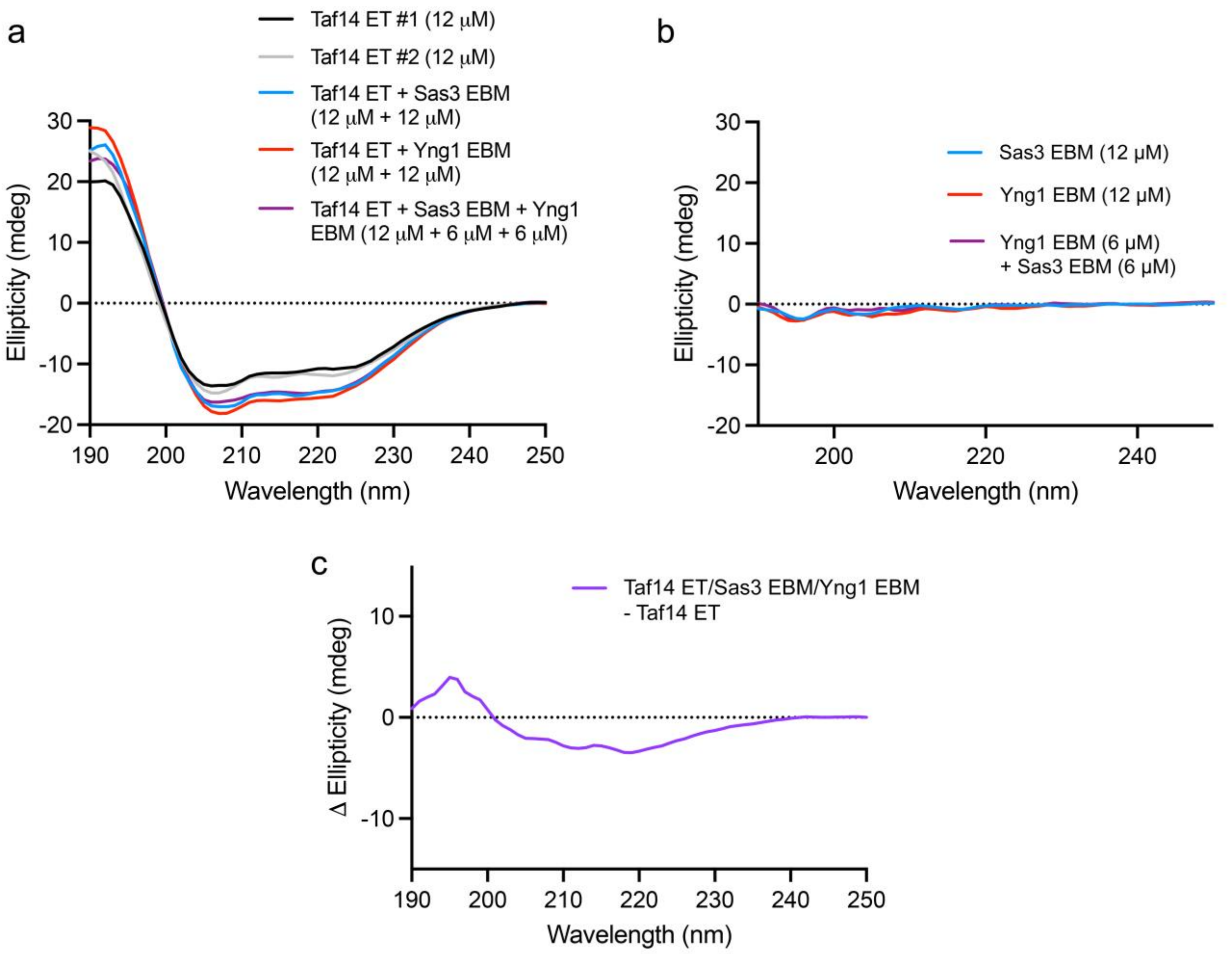

Supplementary Figure 8. (a, b) Circular dichroism (CD) spectra of $Taf14_{ET}$ in the absence and presence of equimolar quantities of $Yng1_{EBM}$ and $Sas3_{EBM}$ are shown in (a) and corresponding CD spectra of EBM peptides are shown in (b). (c) Difference between CD spectra of $Taf14_{ET}$+$Sas3_{EBM}$+$Yng1_{EBM}$ in a 1:0.5:0.5 molar ratio and the apo-state of $Taf14_{ET}$ shows an increase in a β-strand conformation. Related to Figure 6.

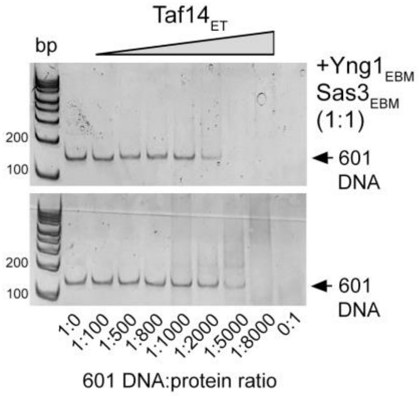

Supplementary Figure 9. EMSAs of 601 DNA in the presence of increasing amounts of $Taf14_{ET}$ with or without the mixture of $Yng1_{EBM}$ and $Sas3_{EBM}$. $Taf14_{ET}$ was either preincubated with $Yng1_{EBM}$ and $Sas3_{EBM}$ at a 1:1:1 molar ratio for a minimum of 1 hour on ice or used without peptides prior to addition of DNA. DNA:protein ratio is shown below gel images. Related to Figure 6.

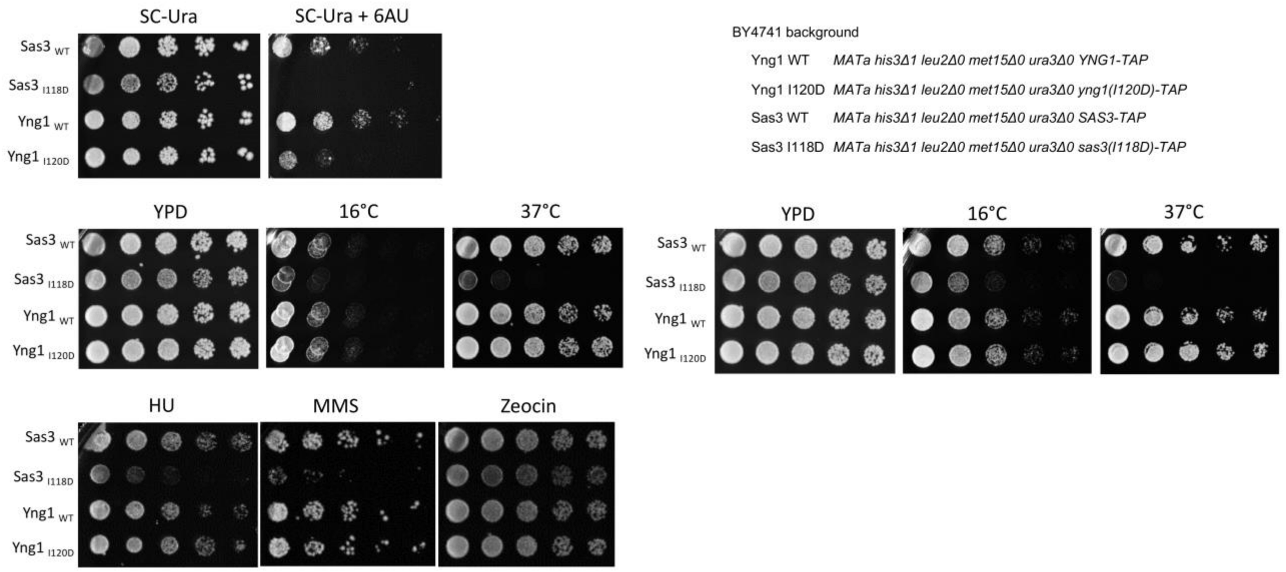

Supplementary Figure 10. (top) 0.5 OD_{600} of the indicated yeast strains were 5-fold serially diluted on synthetic complete (SC) medium lacking Uracil (SC -Ura) or SC -Ura plates supplemented with 100 µg/ml 6-azauracil (6-AU) and grown for 4 days. Yeast strains used in this study are on the right. (middle) 0.5 OD_{600} of the indicated yeast strains were 5-fold serially diluted onto plates containing rich media (YPD) grown at either 30 °C for 3 days, 37 °C for 3 days, or 16 °C for 5 days. (bottom) 0.5 OD_{600} of the indicated yeast strains were 5-fold serially diluted onto YPD-containing plates supplemented with either 100 mM hydroxyurea (HU), 0.03% methanesulfonate (MMS), or 5 µg/ml Zeocin and grown at 30 °C for 3 days. Related to Figure 6.

Supplementary Table 1. Data collection and refinement statistics. Related to Figure 2.

	$Taf14_{ET}$-$Yng1_{EBM}$
Wavelength	
Resolution range	45.84 - 1.933 (2.002 - 1.933)
Space group	P 1 21 1
Unit cell	46.7798 64.6099 64.49 90 101.53 90
Total reflections	55906 (5381)
Unique reflections	28236 (2781)
Multiplicity	2.0 (1.9)
Completeness (%)	99.78 (99.64)
Mean I/sigma(I)	10.92 (4.36)
Wilson B-factor	16.43
R-merge	0.04643 (0.1498)
R-meas	0.06566 (0.2119)
R-pim	0.04643 (0.1498)
CC1/2	0.995 (0.851)
CC*	0.999 (0.959)
Reflections used in refinement	28235 (2781)
Reflections used for R-free	2000 (197)
R-work	0.1869 (0.2123)
R-free	0.2219 (0.2867)
CC(work)	0.946 (0.871)
CC(free)	0.913 (0.742)
Number of non-hydrogen atoms	2883
macromolecules	2552
ligands	0
solvent	329
Protein residues	318
RMS(bonds)	0.005
RMS(angles)	0.83
Ramachandran favored (%)	99.67
Ramachandran allowed (%)	0.33
Ramachandran outliers (%)	0.00
Rotamer outliers (%)	0.00
Clashscore	5.59
Average B-factor	19.63

Statistics for the highest-resolution shell are shown in parentheses.
