# Supplementary material for: Molecular insight into interactions between the Taf14, Yng1 and Sas3 subunits of the NuA3 complex
Source: Nat Commun. 2024 Jun 24;15:5335. doi: 10.1038/s41467-024-49730-y (PMC11196586; doi:10.1038/s41467-024-49730-y)
Supplement: Supplementary file 3 — Description of Additional Supplementary Files [file 41467_2024_49730_MOESM3_ESM.pdf]

Description of Additional supplementary File:

Supplementary Data 1: All pathways enriched in list of genes with promoters co-occupied by Taf14 and Yng1
